# The health gap and HRQoL inequalities in Greece before and during the economic crisis

**DOI:** 10.3389/fpubh.2023.1138982

**Published:** 2023-06-05

**Authors:** John Yfantopoulos, Athanasios Chantzaras, Platon Yfantopoulos

**Affiliations:** ^1^IPOKE Research Institute, MBA National and Kapodistrian University of Athens, Athens, Greece; ^2^MBA National and Kapodistrian University of Athens, Athens, Greece

**Keywords:** HRQoL, health inequalities, health gap, Theil, economic crisis, austerity, Greece, EQ-5D-5L

## Abstract

**Objective:**

The aim of this study was twofold: (i) to assess the health gap among young socio-economic groups generated by the economic crisis in Greece and (ii) to investigate HRQoL (Health Related Quality of Life) inequalities using the Theil index.

**Methods:**

The EQ-5D-5L instrument was administered to a sample of 4,177 young individuals in Greece, mean age 22.3 (±SD 4.8) and 53.8% males, and 46.2% females. The Greek version of the EQ-5D-5L instrument was used in a web-based questionnaire to collect data. Subjects were asked to assess their subjective health status during the economic crisis of 2016 using the EQ-5D-5L instrument, and to recall their health before the crisis of 2009. The health gap was assessed on a Visual Analogue Scale (EQ-VAS), the EQ-5D-5L Index, and the five dimensions of the EQ-5D-5L instrument. Regression analysis was employed to measure the effects of the economic crisis on age, sex, education, and income on the EQ-VAS and EQ-5D-5L. Theil index was used to assess HRQoL inequalities.

**Results:**

The economic crisis brought a significant deterioration in the HRQoL of young Greeks. The EQ-VAS was reduced during the crisis by −10.05% (*p* < 0.001) and the EQ-5D-5L index declined by −19.61% (*p* < 0.001). The prevalence of the health gap in each dimension of the EQ-5D-5L was also significant in terms of deterioration of Mobility [change by 66.8% (*p* < 0.001)], Self-care [change by 61.0% (*p* < 0.001)], Usual activities [change by 97.1% (*p* < 0.001)], Pain/discomfort [change by 65.0% (*p* < 0.001)], and Anxiety/depression [change by 70.5% (*p* < 0.001)]. Significant reductions in EQ-5D-5L indices were also associated with greater inequalities in the distribution of health among age, gender, income, and educational groups. The EQ-5D-5L health gap among the poor was much greater (0.198), in comparison to richer (0.128) classes. Similar gaps were also found in terms of educational inequalities. The EQ-5D-5L health gap among those with primary education was 0.211, whereas for those with tertiary education it was 0.16. The Theil index indicated an increase in income-related HRQoL inequalities by 222.3% for the EQ-5D-5L index and by 124.2% for the EQ-VAS. The effects of demographic and socioeconomic variables on the EQ-VAS were found statistically significant: sex (*p* < 0.05), age (*p* < 0.001), education (*p* < 0.001), and income (*p* < 0.001).

**Conclusion:**

The EQ-5D-5L instrument appears to be a powerful tool in assessing the health gap and the HRQoL inequalities among young people in Greece. The findings indicate the importance of developing effective health policies to combat inequalities and mitigate the impact of austerity measures on the quality of life of the young.

## Introduction

The notion of the health gap was initially discussed by Sir Michael Marmot (1–3) and was further adopted by the World Health Organization as a key public health issue to combat health inequalities. The WHO set a Commission on the Social Determinants of Health to investigate health policies for “closing the gap in a generation” (1). The majority of this literature on health inequalities is based on macro-epidemiological indicators describing the existing regional and national differences in life expectancy and healthy life years among European nations as well as among professional and socio-economic groups (4–6).

The health inequalities among the European Nations were further aggravated during the economic crisis of the 2010s (7). The impact of economic recessions on health has been widely investigated in the literature of public health and health economics with controversial results. The majority of studies used a combination of aggregate mortality and macroeconomic data based on time series. Pritchett and Summers (8), argued that “wealthier is healthier,” and Brenner indicated that economic recessions have a negative impact on health (9, 10). Gerdtham and Johannesson (11) found that male mortality (but not female mortality) increased significantly during the economic recession in Sweden.

In a recent study, McCartney, Fenton, Minton, Fischbacher, Taulbut, Little, Humphreys, Cumbers, Popham and McMaster (12) used panel data for 37 high income countries over the period 2000–2019 and found that austerity adversely impacted on life expectancy and age specific mortality across England, Estonia, Iceland, Scotland, Slovenia, and the USA. Generally, females were affected more than males.

In our analysis we expand the existing literature in two ways (i) we use disaggregated individual data based on surveys and (ii) we assess the effects of economic recession on health by using more sensitive indicators based on health-related quality of life (HRQoL) rather than using mortality or morbidity data (5). We investigate the effects of the economic crisis in Greece before (2009) and during (November 2015 to April 2016) by using the EQ-5D-5L instrument. Reviewing the EQ-5D literature, we have found a limited number of publications using the EQ-5D instrument for measuring the effects of the economic crisis on the population’s quality of life (13).

Given the shortage of knowledge in this area of research, the purpose of this study is to investigate the application of the Greek version of the EQ-5D-5L instrument in measuring the effects of the economic downturn on the health-related quality of life (HRQoL) of the young Greek Population.

### The Greek crisis

The economic crisis in Greece lasted for 10 years (2009–2019) with devastating effects on the health and the economic status of the people and particularly in the younger generations. Before the crisis, and during the 2000s, Greece enjoyed prosperous economic growth with an annual increase in GDP fluctuating around 4%, whereas the corresponding growth of the average EU-28 was just about 2% (14).

By the end of 2009 the Greek economy’s macroeconomic indicators, in terms of competition, investment, budget deficit and general government debt were the most ominous in relation to the rest of the EU. The budget deficit was 15.4% of GDP and the debt was 127% of GDP with an indication of further increasing trends. The European Commission, (EC) the European Central Bank (ECB) and the International Monetary Fund (IMF), the so-called “Troika,” undertook the responsibility to provide three rescue packages to Greece (15, 16).

The first in May 2010 was worth €110 billion, the second in February 2012 amounting to €130 billion, and the third in August 2015 was worth €86 billion euros. The signed terms of these bailouts included a series of fiscal and economic measures.

Key reforms were expected to be undertaken concerning the health sector, the labor market, the reduction of public expenditure, the fight against corruption and the underground economy, the control of health expenditure, and the implementation of three austerity packages. Some of these reforms had been on the political agenda of leading parties in Greece for decades. However, due to high political risk and fears of trade union opposition, they had never been implemented until then. The Greek economic crisis imposed substantial adverse effects on Greek society in general and particularly on the population’s health (13, 17–21). More specifically, over the period 2009–2019, GDP was reduced by 20%, health expenditure declined by 30%, and unemployment increased by 253%. Special reference should be made to youth unemployment which, starting from a level of 21.7% in 2008 increased all the way to 58.2% in 2013 which is by far the highest in the European Union and the OECD (Organization of Co-Operation and Development) (21). Greek youth unemployment was exacerbated during the 10 years of economic crisis by austerity measures causing the “economic massacre of the younger generation” (OECD 2015). Youth unemployment had also caused a mass exodus of the young population from Greece to other EU-28 countries. According to OECD data (OECD 2015), the proportion of Greek Citizens’ satisfaction with the health care system substantially decreased from 52% in 2007 to 35% in 2015, which is the lowest value among OECD countries (22).

The effects of the financial crisis on health and the health care in Greece have been discussed by several authors (23). Simou and Koutsogeorgou (23) provide evidence on the deleterious effects of the economic crisis on the Greek people. They carried out a systematic literature review for the period January 2009 to March 2013 and referred 39 studies. The findings highlighted the implications of public health expenditure cuts and relevant memorandum policies, on the increasing rates of mental health, suicides, and the deterioration of self-rated health.

## Methods

### Study design

During the period from November 2015 to April 2016, a web-based survey was launched targeting the young Greek population, aged 18–39 years. The study focused on the investigation of the HRQoL of the young population because they were seriously affected by the high unemployment rates in comparison to the rest of the Greek population. Before the economic crisis and throughout the 2000’s youth unemployment fluctuated on the average around 25%. In the peak of the crisis in 2013 reached 58.2% and remained high at 47.1% in 2016 (year of launching the survey). The total unemployment rate was less than 10% before the crisis (during the 2000’s) and fluctuated around 23.5% in 2016. The rational of our study was also supported by Drydakis (24). He used longitudinal labor market data over the period 2008–2013 and he highlighted the statistically negative relationship between unemployment and self-reported health as well as mental health. For our analysis, it was considered important to investigate further the effects of the crisis by focusing on the measurement of HRQoL and assessing the magnitude to health inequalities and the health gaps across sociodemographic groups. The study was designed in such a way as to ensure representativeness of the sample as much as possible with respect to age, and sex. Obtaining access to diverse groups that may represent different levels of health and quality of life profiles was crucial to the design of the study. The collaboration between the University of Athens and the Hellenic Statistical Authority (ELSTAT) was fundamental for the web-design and the launching of questionnaires. The structured questionnaire used in the current study included three main components: (1) demographic factors, (2) Socio-economic, and (3) the EQ-5D-5L questionnaire. Data were collected by the research team at the University of Athens using a web-based survey and a convenient sampling method. The sample consists of 4,177 young people, with 2,246 males (53.8%) and 1931 females (46.2%). The mean age of the respondents was 22.3 (±SD 4.8 years). The subjects were asked to administer a self-reporting questionnaire, regarding their socio-demographic profile, income, education, health status, and quality of life. The EQ-5D-5L instrument was employed to obtain information on self-perceived health “before” the burst of the crisis in the year 2009 and “during” the crisis in the year 2016. The respondents were initially asked to assess their “current” health state in a VAS scale and in the five dimensions of the EQ-5D-5L instrument. Subsequently, the respondents were asked to recall their health state before the crisis (by making reference in the year 2009). In the preparation of the study design, special effort was taken to reduce, or even eliminate the effect of all possible implications related to memory. We provided some guidelines to the respondents to recall their economic and social conditions in the year 2009 and to assess their health and quality of life by considering these the socio-economic factors. The HRQoL values were calculated for different gender, demographic, and socio-economic groups. Finally, comparisons were established by distinguishing the states of health “before the crisis” (2009) and “during the crisis” (2016).

### EQ-5D-5L

Over the last 30 years, the EuroQol instrument (EQ-5D-3L) has been developed by the EuroQol group (25, 26) and it has been widely applied as a generic preference-based instrument for the measurement of populations’ health across the world (27). After several years of investigation and experimentation with pilot studies, the EuroQol group introduced a 5-level EQ-5D questionnaire that expanded the range of responses for each dimension from three to five levels (28). Several studies across Europe, the USA, and Canada have indicated the improved psychometric properties of 5 L over 3 L in terms of the reduced ceiling and floor effects, increased reliability, and discriminative ability between different levels of health (29).

In Greece, the EQ-5D-3L was translated and validated by Yfantopoulos (30) and the 5-level version in 2017 (31) and has been widely used for both population and disease-specific studies. Following the international experience, the EQ-5D-5L version was applied to several clinical and population studies (31–33).

The EQ-5D-5L instrument consists of two components: (1) The EQ-5D-5L descriptive system and (2) the EQ-VAS. The EQ-5D descriptive system consists of five dimensions of health: (i) Mobility, (ii) Self-care. (iii) Usual activities, (iv) Pain/discomfort, and (v) Anxiety/Depression. Each dimension is rated in a five-level scale ranging from No-Problems to Severe Problems. The level of functioning classifies respondents into unique health states (Health Profile) that are often reported as a five-digit vectors ranging between 11,111 (full health) to 55,555 (worst health). This classification leads to 5^5^ = 3,125 distinct health states (13). A single utility score can be obtained by applying societal value sets derived from population-based valuation studies. These studies have been conducted in a great number of countries in an attempt to reflect cultural adaptation of country related societal values. The health utilities for this study are based on the UK tariffs which have been applied to the Greek setting (31). The EQ-VAS scale records the respondents’ self-rated health on a 20 cm visual analogue scale with endpoints labeled “the best imaginable health” (100) and “the worst imaginable health” (0). The VAS provides a direct subjective evaluation of the respondents’ health before the crisis, indicated as **EQ-VAS**_**Before**_ and during the crisis indicated as **EQ-VAS**_**During**_. The EQ-5D descriptive system was also used in order to explore the health profile for each dimension of health indicating a self-assessed health state “Before” and “During” the crisis. These health states have been converted into a weighted health index by applying the EuroQol website scores. The EQ-5D preference weights were subsequently elucidated from general population valuation studies. The weights lie on a scale ranging from 0 = death, to 1 = perfect health. In the current study, population weights were used to convert the EQ-5D index score to represent utility values before (**EQ-5D-5L-Index**_**Before**_) and during the crisis (**EQ-5D-5L-Index**_**During**_).

### Statistical analysis

Mean values for the EQ-VAS and EQ-5D-Index scores were estimated for different demographic and socioeconomic groups. Statistical significance was set at a probability level of *p* < 0.001, to ensure the highest level of confidence in the probabilistic significance test conducted throughout the study.

Four multivariable linear regression models were specified to identify the marginal effects of the crisis on quality-of-life indicators. The dependent variables were EQ-5D-5L-Index, and EQ-VAS and the independent variables included the five dimensions of mobility (MOB), self-care, (SEL CAR) usual activities (US.AC), pain/discomfort, (PA-DIS) anxiety/depression (AN-DEP) for models 1 and 2, and four additional sociodemographic variables, (Age, Sex, Education and Income) were included in the models 3 and 4. The sociodemographic variables used in the models 3 and 4 were the following: Sex (Male/Female), Age (Continuous variable for young population from 18–39 years), Education (Primary, Secondary, Tertiary), Income (Household income was equivalized with the square root of household size).

The four specified models are presented below, and the results are portrayed in Tables 4, 5 in the results section.

### Before crisis effects

**MODEL 1: EQ-5D-5L-INDEX**_**BEFORE**_ **= F (MOB, SEL CAR, US.AC, PA-DIS, AN-DEP)**

**MODEL 2: EQ-VAS**_**BEFORE**_ **= F (MOB, SEL CAR, US.AC, PA-DIS, AN-DEP)**

### During crisis effects

**MODEL 3: EQ-5D-5L-INDEX**_**DURING**_ **= F (MOB, SEL CAR, US.AC, PA-DIS, AN-DEP Age, Sex, Education, Income)**

**MODEL 4: EQ-VAS**_**DURING**_ **= F (MOB, SEL CAR, US.AC, PA-DIS, AN-DEP Age, Sex, Education, Income)**

The statistical specifications of the above models were tested using ordinary least square methods.

### HRQoL inequality indexes

In the health economic literature, the first comprehensive approach to measuring health inequalities was published by Wagstaff et al. (34). They critically assessed the various measures used to evaluate trends and cross-country differences in socio-economic inequalities in health. Subsequently, Kunst, Mackenbach and World Health Organization. Regional Office for Europe (35) and Mackenbach and Kunst (36) published a more detailed analysis of health inequality measures by expanding the previous work of Wagstaff et al., and by presenting some indicative examples using European data. In 1990 and 2000, some researchers from the WHO and the Eurostat suggested that emphasis should be given to individual data and not to aggregate analysis (37–39). Subsequently there have been several attempts in the epidemiological and health economic literature highlighting the properties of different measures of health inequalities (40, 41). In our analysis we focus on individual data in an attempt to assess the magnitude of health-related quality-of-life inequalities across socioeconomic groups in Greece. We make use of the Theil index (42) in order to assess the sensitivity in the upper and lower bound in the distribution of health-related quality of life.

### Theil index

Theil developed an index derived from Shannon’s measure of information entropy to measure the unfairness of income distribution (42). The formula for computing the Theil index is as follows (43):


$$T=\frac{1}{n}\overset{n}{\underset{i=1}{\sum }}\frac{h_{i}}{\mu }\ln\left(\frac{h_{i}}{\mu }\right)$$

Where 1/n is an individual’s I population share, hi/μ is the ratio of the individual’s health status to the population average. The Theil index ranges between 0 (absence of inequality) and ln (n) (maximum inequality).

When the population of individuals can be arranged into j (socioeconomic) groups, the Theil index can be decomposed to two parts (additive decomposability): the between-group inequality and a weighted average of within-group inequality:


$$T=T_{B}+T_{W}=\left[\overset{J}{\underset{j=1}{\sum }}p_{j}\frac{{\bar{h}}_{j}}{\mu }\ln\left(\frac{{\bar{h}}_{j}}{\mu }\right)\right]+\left[\overset{J}{\underset{j=1}{\sum }}p_{j}\frac{{\bar{h}}_{j}}{\mu }T_{j}\right]$$

Where TB stands for the absolute value of between-group inequality element, TW denotes the absolute value of within-group inequality and Tj component is the inequality in health status within group j, which is weighted by group j’s share of the total health status. The between-group inequality component can be interpreted as a socioeconomic health inequality measure.

## Results

The results are based on a web survey, conducted at the School of Economics and Political Science of the University of Athens over the period from November 2015 to April 2016. As many as 4,177 young people completed the EQ-5D-5L instrument of which 2,246 males (53.8%) and 1931 females (46.2%). The mean age of the respondents was 22.3 (± SD 4.8 years). The results of the analysis indicate a significant impact of the Greek economic crisis in the deterioration of the health status and quality of life of the young Greek Population. Table 1 provides a summary of the average EQ-5D-5L and the EQ-VAS values before and during the crisis as well as the changes in prevalence of the five dimensions of the EQ-5D-5L. Absolute and relative changes (in %) describe the significant deterioration of HRQoL before and during the economic crisis. In the EQ-5D-5L index the absolute change was −0.164 and the relative change −19.61%. In the EQ-VAS the corresponding changes were −8.76 and −10.05%. The *p* values for these estimates as well as for the prevalence of the EQ-5D-5L dimensions before and during the economic crisis were all significant at *p* < 0.001. In particular the *p* values for the within-groups differences were the following: (i) EQ-5D-5L index (*p* < 0.001), (ii) EQ-VAS (*p* < 0.001), and then for each dimension of EQ-5D-5L were all statistically significant: (i) Mobility (*p* < 0.001), (ii) Self-Care (*p* < 0.001), (iii) Usual Activities (*p* < 0.001), (iv) Pain/Discomfort (*p* < 0.001), and (v) Anxiety/Depression (*p* < 0.001).

**Table 1:** The EQ-5D-5L and EQ-VAS mean values and prevalence of the five EQ-5D-5L dimensions.

	Before crisis	During crisis	Absolute change	Relative change (%)	*p*-value
EQ-5D-5L index	0.838	0.673	−0.164	−19.61	<0.001
EQ-VAS	87.13	78.37	−8.760	−10.05	<0.001
Prevalence of problems %					
Mobility	25.3	42.2	16.9	66.8	<0.001
Self-care	25.1	40.4	15.3	61.0	<0.001
Usual activities	27.6	54.4	26.8	97.1	<0.001
Pain/discomfort	28	46.2	18.2	65.0	<0.001
Anxiety/depression	42	71.6	29.6	70.5	<0.001

Tables 2, 3 present the EQ-5D-5L and EQ-VAS estimates before and during the crisis. The analysis is based on the socioeconomic determinants of health inequalities by distinguishing three groups: (i) Education inequalities, (ii) Household income inequalities, and (iii) Subjective income inequalities. Mean values and 95% confidence intervals between lower and upper bounds are presented in Tables 2, 3.

**Table 2:** Socio-economic results for the EQ-5D-5L before and during the crisis.

EQ-5D-5L index before crisis				EQ-5D-5L index before crisis		
	Mean	95% Confidence interval		Mean	95% Confidence interval	
		Lower bound	Upper bound		Lower bound	Upper bound
Overall	0.83774	0.8318	0.8436	0.67348	0.6649	0.6820
Education level						
Primary	0.80925	0.7545	0.8639	0.59779	0.5255	0.6700
Secondary	0.8422	0.8245	0.8598	0.6407	0.6099	0.6714
Tertiary	0.83789	0.8316	0.8441	0.67788	0.6689	0.6868
Household income level						
Less than 500 €	0.76907	0.7366	0.8015	0.57147	0.5318	0.6110
500–999 €	0.82618	0.8104	0.8419	0.62228	0.5981	0.6463
1.000–1.499 €	0.83706	0.8234	0.8506	0.65283	0.6314	0.6741
1.500–1.999 €	0.84237	0.8280	0.8566	0.67427	0.6522	0.696
2.000–2.999 €	0.84711	0.8340	0.8601	0.70004	0.6814	0.7186
> = 3.000 €	0.85755	0.8443	0.8707	0.72979	0.7122	0.747
Subjective household income						
Very bad	0.79285	0.7547	0.8309	0.54639	0.4927	0.6000
Bad	0.81403	0.7975	0.8304	0.59358	0.5688	0.6182
Average	0.83302	0.8246	0.8413	0.66825	0.6561	0.6803
Good	0.85891	0.8485	0.8692	0.72669	0.7121	0.7412
Very good	0.8691	0.8410	0.8971	0.76564	0.7310	0.8002

**Table 3:** Socio-economic results for the EQ-VAS before and during the crisis.

EQ-VAS before crisis				EQ-VAS during crisis		
Overall	Mean	Lower	Upper	Mean	Lower	Upper
	87.13	86.71	87.56	78.37	77.78	78.96
Education level						
Primary	79.58	75.15	84	64.93	59.71	70.14
Secondary	85.23	83.64	86.82	72.46	70.01	74.9
Tertiary	87.45	87	87.89	79.16	78.56	79.76
Household income						
Less than 500 €	83.56	81.49	85.63	72.47	69.8	75.14
500–999 €	84.76	83.54	85.99	73.71	72.01	75.41
1.000–1.499 €	86.51	85.45	87.56	75.88	74.33	77.44
1.500–1.999 €	88.29	87.25	89.34	78.87	77.27	80.48
2.000–2.999 €	87.99	87.04	88.94	80.91	79.69	82.13
> = 3.000 €	89.23	88.43	90.04	82.43	81.34	83.51
Subjective household income						
Very bad	84.55	82.25	86.84	72.12	68.64	75.6
Bad	85.14	83.99	86.3	71.39	69.64	73.14
Average	86.8	86.16	87.44	78.76	77.92	79.59
Good	88.67	87.94	89.39	80.92	79.88	81.95
Very good	89.58	87.81	91.34	85.49	83.45	87.53

### EQ-VAS and EQ-5D-5L health -gap

**Figure 1:**
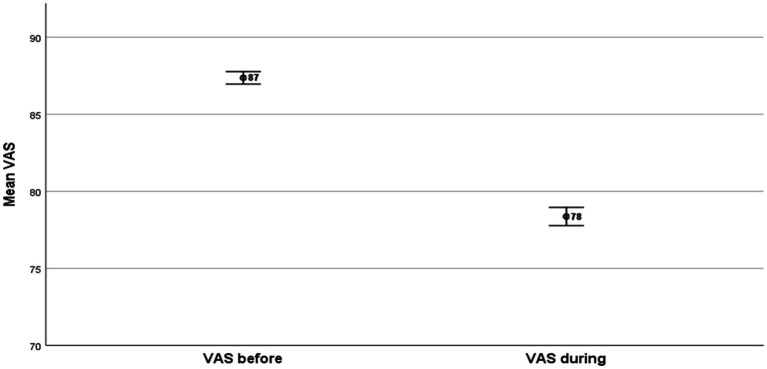
EQ-VAS health gap.

The respondents declared statistically significant higher mean levels of EQ-VAS before the economic crisis in comparison to those declared during the crisis (Figure 1). The EQ-VAS mean value before the crisis was **EQ-VAS**_**Before**_ = 87 and the corresponding value during the crisis was **EQ-VAS**_**During**_ = 78. The EQ-VAS health gap was = 9 (*p* < 0.001).

**Figure 2:**
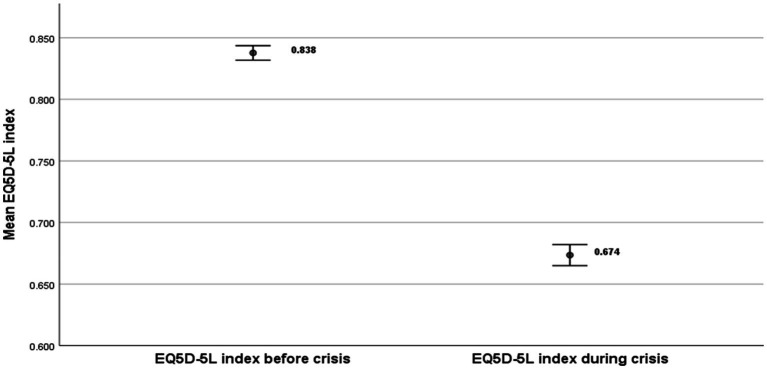
EQ-5D-5L health index gap.

The EQ-5D-5L mean values indicated a larger gap in relation to EQ-VAS because they consider all the relevant five EQ-5D-5L dimensions affected by the crisis (Figure 2). The mean value before the crisis was EQ-5D-5L_Before_ = 0.838 and the corresponding value during the crisis was EQ-5D-5L_During_ = 0.674. The EQ-5D-5L health gap was = −8.76 (*p* < 0.001).

**Figure 3:**
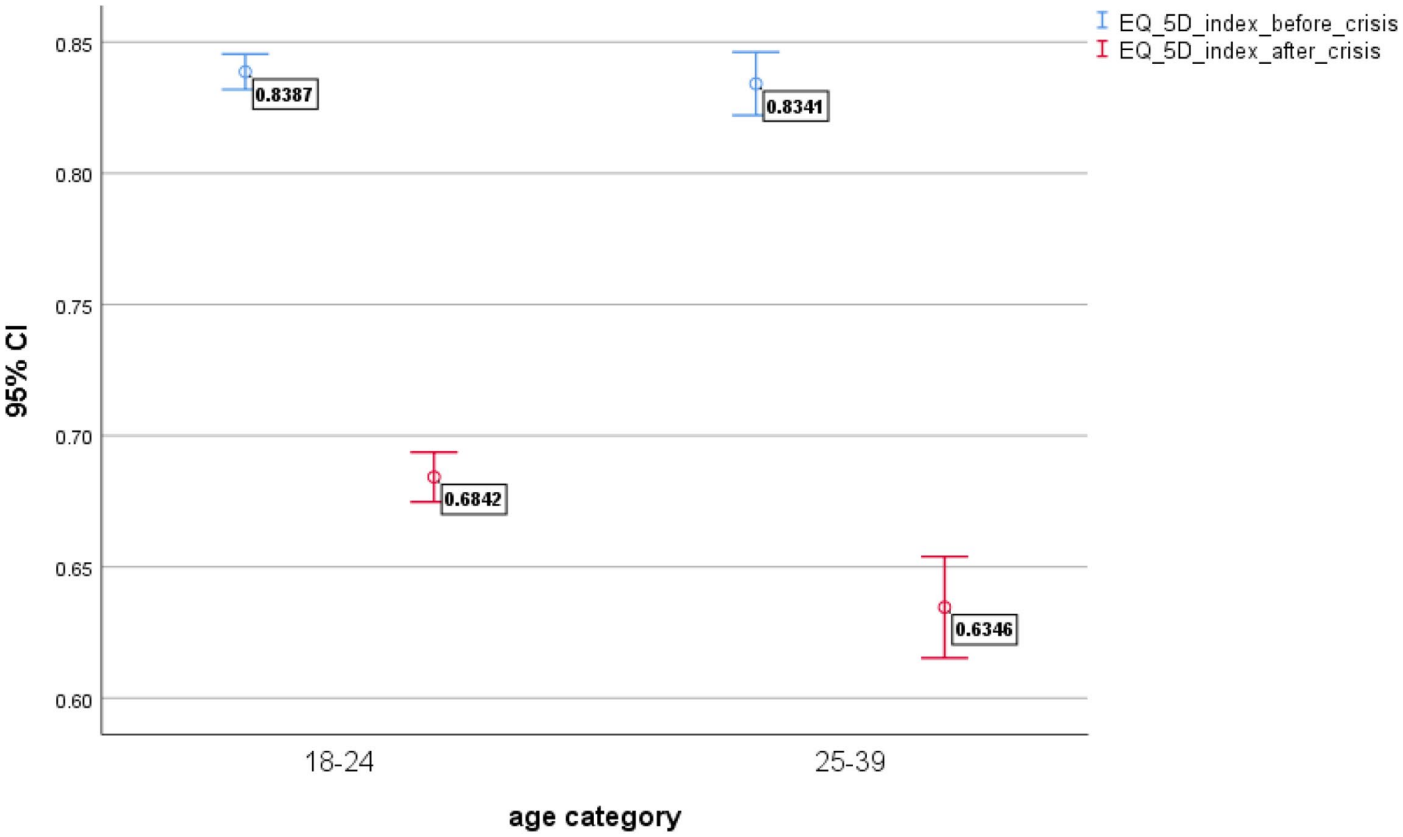
EQ-5D-5L health gap by age category.

The effects of the economic crisis are better portrayed in the distribution of health utilities by age group (Figure 3). We distinguish between two groups of youths: those who are aged 18–24 and those who are aged 25–39.

The strong effects of the crisis on the youth are shown in Figure 3 with a larger health gap in the age group 25–39 (health gap = 0.20) (*p* < 0.001) in relation to the age group 18–24 (health gap = 0.15) (*p* < 0.001).

**Figure 4:**
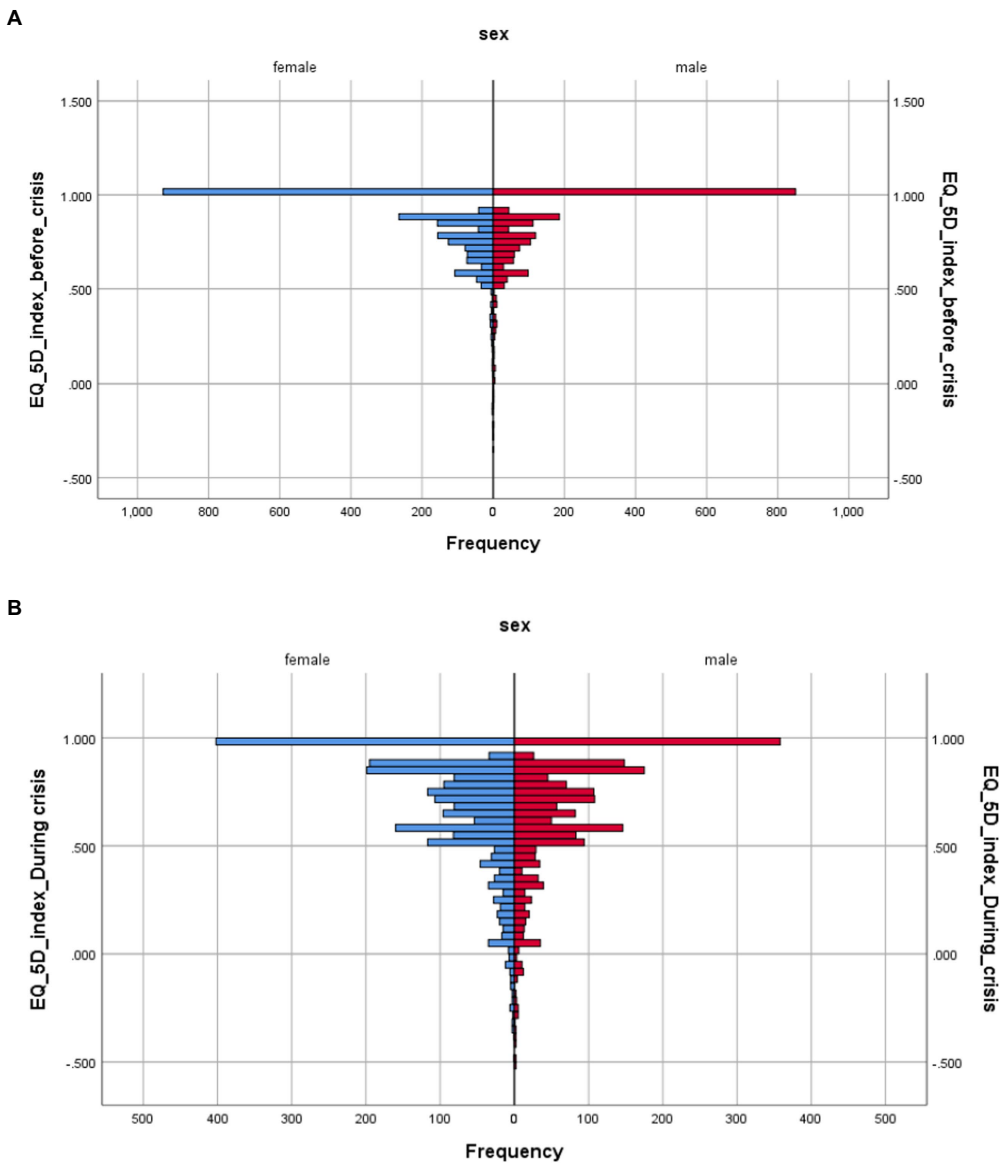
**(A,B)** EQ-5D-5L health distribution before and during the economic crisis by sex.

Gender health inequalities is also an important area for public health and relevant policy implications. In Figures 4A,B we present the distribution of health before and during the economic crisis by sex. The effects of the crisis are better described in Figure 4B with an extended distribution of health utilities toward lower levels of health utility values.

**Figure 5:**
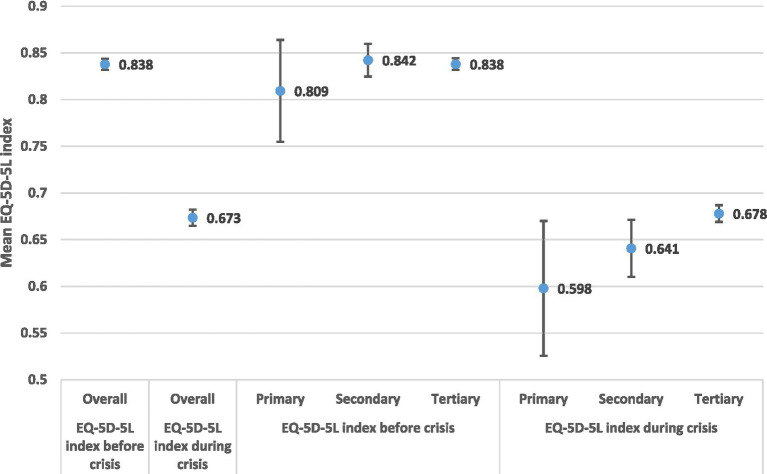
EQ-5D-5L health gap by educational levels.

Education-related health quality of life inequalities are shown in Figure 5. The EQ-5D-5L health gap in the lower educational level is higher in relation to tertiary education. The EQ-5D-5L- health gap _primary_ = 0.211, i.e., (EQ-5D-5L Primary _Before_ = 0.809 minus EQ-5D-5L Primary _During_ = 0.598). The health gap is maintained in the secondary education and becomes smaller in the tertiary education. The EQ-5D-5L- health gap _tertiary_ = 0.16, i.e., (EQ-5D-5L Tertiary _Before_ = 0.838 minus EQ-5D-5L Tertiary _During_ = 0.678).

**Figure 6:**
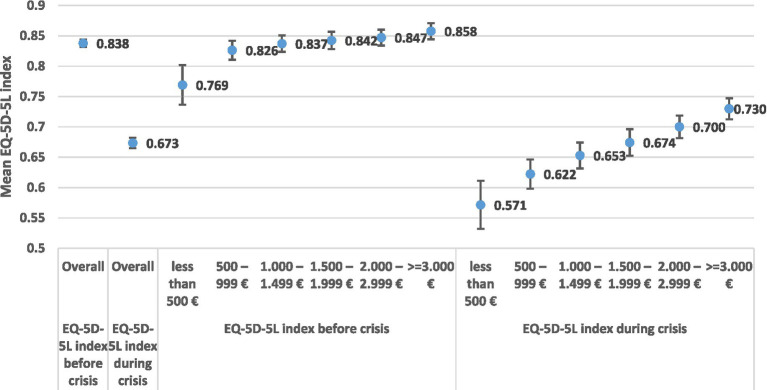
EQ-5D-5L health gap by income groups.

**Figure 7:**
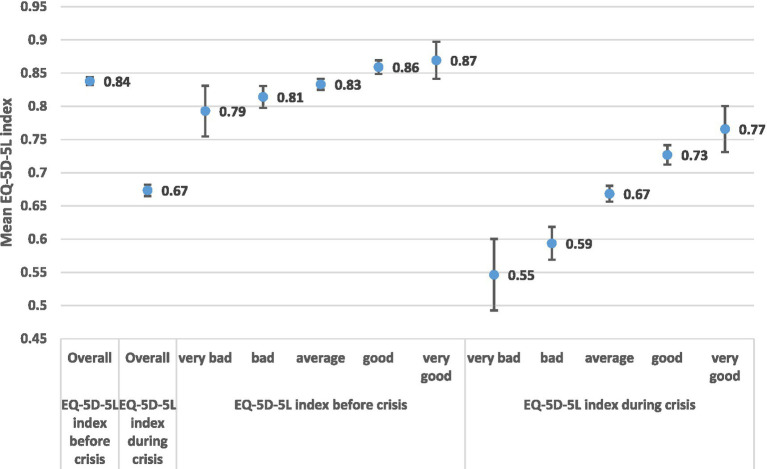
EQ-5D-5L health gap by subjective household income groups.

Income related health quality of life inequalities are presented in Figures 6, 7. Income is often difficult to capture and report adequately, because of underreporting problems. In our analysis we tested income inequalities using a numeric income distribution based on six household groups (Figure 6) and a subjective income distribution by distinguishing five income groups (very bad, bad, average, good, very good). It is interesting to note that the subjective income distribution (Figure 7) provided higher health gaps in comparison to the numeric income distribution (Figure 6).

Focusing our discussion on Figure 6 the EQ-5D-5L- Health Gap _poor (500<)_ = 0.198, i.e., (EQ-5D-5L _Poor Before (500<)_ = 0.769 minus EQ-5D-5L _Poor During (500<)_ = 0.571). The health gap among the rich is EQ-5D-5L- health gap _Rich (>3000)_ = 0.128, i.e., (EQ-5D-5L _Rich (>3000) Before_ = 0.858 minus EQ-5D-5L _Rich (>3000) During_ = 0.730).

In the case of subjective income distribution (Figure 7) the estimated health gap is much higher in the lower income classes in comparison to higher classes. EQ-5D-5L- health gap _poor (very bad)_ = 0.247, i.e., (EQ-5D-5L _Poor Before (very bad)_ = 0.793 minus EQ-5D-5L _Poor During (very bad)_ = 0.546). The health gap in the higher levels of subjective income is EQ-5D-5L- health gap _Rich (very good)_ = 0.103, i.e., (EQ-5D-5L _Rich (very good) Before_ = 0.869 minus EQ-5D-5L _Rich (very good) During_ = 0.766).

#### Multivariable linear regression models

The empirical results of the multivariable linear regression models are shown in Table 4 for the models 1 and 2, presenting estimates for the period before the economic crisis, and Table 5 for the models 3 and 4 describing estimates for the period during the economic crisis. Table 4 presents the marginal effects of the five dimensions of the EQ-5D-5L instrument, (Mobility, Self-Care, Usual Activities, Pain Discomfort and Anxiety Depression) on the dependent variables EQ-5D-5L Index and EQ-VAS. The explanatory powers of the estimated models 1 and 2 are presented by the coefficient of determination (*R*^2^).

**Table 4:** Regression results for the EQ-5D-5L and EQ-VAS before crisis.

	Model 1: dependent variable EQ-5D-5L index before			Model 2: dependent variable EQ-VAS before		
	*B*	Std. Error	Sig	*B*	Std. Error	
(Constant)	1.318	0.002	*p* < 0.001	98.696	0.573	*p* < 0.001
Mobility before	−0.052	0.002	*p* < 0.001	−2.002	0.447	*p* < 0.001
Self-care before	−0.072	0.002	*p* < 0.001	0.157	0.437	*p* < 0.720
Usual activities before	−0.029	0.002	*p* < 0.001	−1.128	0.424	*p* < 0.008
Pain discomfort before	−0.097	0.001	*p* < 0.001	−2.279	0.372	*p* < 0.001
Anxiety depression before	−0.088	0.001	*p* < 0.001	−2.754	0.298	*p* < 0.001
R squared	0.929			0.107		

**Table 5:** Regression results for the EQ-5D-5L and EQ-VAS during crisis.

	Model 3: dependent variable EQ-5D-5L index during			Model 4: dependent variable EQ-VAS during		
	*B*	Std. Error	Sig	*B*	Std. Error	Sig
(Constant)	1.292	0.009	*p* < 0.000	98.407	2.149	*p* < 0.000
Mobility during	−0.052	0.002	*p* < 0.000	−1.606	0.485	*p* < 0.001
Self-care during	−0.056	0.002	*p* < 0.000	0.231	0.495	*p* < 0.641
Usual activities during	−0.038	0.002	*p* < 0.000	−1.566	0.396	*p* < 0.000
Pain discomfort during	−0.081	0.002	*p* < 0.000	−3.161	0.393	*p* < 0.000
Anxiety depression During	−0.095	0.001	*p* < 0.000	−3.164	0.315	*p* < 0.000
Sex	−0.005	0.002	*p* < 0.047	−1.148	0.574	*p* < 0.046
Age	0.000	0.000	*p* < 0.597	−0.367	0.061	*p* < 0.000
Education	0.003	0.002	*p* < 0.054	1.720	0.375	*p* < 0.000
Income	0.000	2E-06	*p* < 0.075	0.002	0.001	*p* < 0.001
R squared	0.935			0.210		

##### Model 1

Around 93% of the variance of the EQ-5D-5L Index is explained by the estimated model 1. This is expected because the dependent variable EQ-5D-5L Index is directly composed of the five dimensions of the EQ-5D-5L instrument In the model 1 all the estimated coefficients are statistically significant at *p* < 0.001 (Table 4). The highest marginal effects are for pain/discomfort (− 0.097) and anxiety/depression (−0.088).

##### Model 2

In the case of model 2 the coefficient of determinations (*R*^2^) is only *R*^2^ = 0.107 indicating a much lower explanation of the variance of EQ-VAS in comparison to EQ-5D-5L Index. Anxiety/depression is statistically significant (*p* < 0.001) with the highest marginal effects (−2.754) in comparison to the rest variables included in the model 2.

Table 5 presents the results for models 3 and 4. Both models 3 and 4 extend the analysis of the 5 dimensions of the EQ-5D-5L instrument as explanatory variables and include the effects of sociodemographic factors such as: sex, age, education, and income.

##### Model 3

The coefficient of determination (*R*^2^ = 0.935) indicates a slight increase in the explanatory power of the model 3 in comparison to model 1. Around 94% of the variance of the EQ-5D-5L Index is explained by the specified model. However, despite the high (*R*^2^) value the statistical significance of the socio-economic variables is limited to around *p* < 0.05 for sex and education.

##### Model 4

The coefficient of determination (*R*^2^ = 0.21) for model 4, increased in comparison to model 2 (*R*^2^ = 0.107). The sociodemographic variables of age, education and income are significant at *p* < 0.001. Anxiety/depression in model 4 is also significant at *p* < 0.001 presenting a much higher effect (−3.164) on EQ-VAS during the crisis period, in comparison to the corresponding effect of the pre-crisis period (−2.754). This indicates the psychological effects of the crisis on the young population.

### Theil index

Theil index is a sensitive indicator in describing the distribution of the HRQoL among individuals and socio-economic groups. Applying Theil’s methodology to our data we found that the overall income related health inequality increased substantially (Table 6). The EQ-5D-5L Theil index before the crisis was EQ-5D-5L (Theil) _Before_ = 0.032 and during the crisis was EQ-5D-5L (Theil) _During_ = 0.104 (% relative increase by 222%). The EQ-VAS (Theil) index provided smaller estimates. The EQ-VAS (Theil) _Before_ = 0.016 and EQ-VAS (Theil) _During_ = 0.036 (% relative increase by 124%). The decomposition analysis revealed a substantial increase by 104% in health inequalities (HRQoL) attributed to income (between groups change) (Table 6).

**Table 6:** Theil index for income related HRQoL inequalities.

Income-related HRQoL inequalities					
	Theil’s index	Theil’s index between groups	Theil’s index within groups	% of Theil between groups in total	% of Theil within groups in total
EQ-5D-5L index before crisis	0.0322	0.0003	0.0320	0.90%	99.10%
EQ-5D-5L index during crisis	0.1039	0.0019	0.1020	1.83%	98.17%
Absolute change	0.0717	0.0016	0.0701	0.93%	−0.93%
Relative change (%)	222.3%	557.8%	219.3%	104.1%	−0.9%
VAS before crisis	0.0159	0.0002	0.0157	1.21%	98.80%
VAS during crisis	0.0355	0.0009	0.0346	2.58%	97.42%
Absolute change	0.0197	0.0007	0.0190	1.37%	−1.38%
Relative change (%)	124.2%	379.6%	121.1%	113.9%	−1.4%

## Discussion

The purpose of this study was to contribute to the literature of health inequalities in two ways: (i) by investigating the health gap generated by the economic crisis on health-related quality of life of young Greek people and (ii) assessing the magnitude of between and within health inequalities by applying Theil’s index methodology on the EQ-5D-5L and EQ-VAS indexes. A great part of the public health and health economic literature on health inequalities focused on macro-epidemiological and macro- economic analysis using time series aggregate data (39, 44).

A common conclusion of these studies is the fact that despite the generosity of the welfare state in increasing social and health expenditure, health inequalities remained “unbridged” between European nations, regions, and socio-economic groups (45–50). There have been various success and failures of the different welfare states across the Scandinavian, Western, Northern, and Southern European typologies in assessing health inequalities. Although the vast part of the literature focused on health outcome data based on objective or subjective health indicators very little research has been conducted in measuring health inequalities using the EQ-5D instrument. Furthermore, the effects of the 2010’s economic crisis on the health status of the European population have been investigated at some length but limited research has been carried out in the health-related quality of life. The economic downturn in 2009 significantly affected the economy, the society, and the health system. GDP growth decreased on average in the EU-27 Member States by 4.3% imposing a substantial decrease in health spending. In the period 2000–2009 the mean annual growth of *per capita* health expenditure in the OECD countries was 4.6% and the corresponding rate had fallen down to 0.6% by 2011 (51). Greece was the only OECD country exhibiting devastating reductions in health expenditures: −2.9 percent in 2009, −11.4 percent in 2010, and −12.2 percent in 2012. During the 2009 to 2014 crisis period, Greek health spending was cut by 34 percent on aggregate, while the corresponding decrease in other OECD countries was almost insignificant. Greece witnessed a much harder economic crisis than any other European Member State, with three memorandums signed: the first in May 2010, worth €110 billion, the second in February 2012 amounting to €130 billion and the third in August 2015 worth €86 billion euros. The main purpose of these financial bailout measures was to rescue the economy from bankruptcy, and reduce as much as possible, the catastrophic effects of the crisis on society and the deterioration of health status of the population. However, the severe austerity measure had a serious negative impact on the health of the Greek population both in terms of life expectancy and years of healthy survival. Life expectancy in Greece increased at a slower rate than many EU countries between 2009 and 2020. While infant mortality remained for many years at lower levels than the European average, it increased significantly in the period during the crisis, surpassing the average of the EU28 Member States. Furthermore, Greece had lost a total of 3.4 years of healthy survival during the period 2006–2016. Hence, the detailed analysis on the impact of the economic crisis on health becomes an important public health policy issue (14, 17, 18, 20, 23, 52).

This paper contributes to the promotion and design of public health policies in Greece in three ways: firstly from a priority agenda setting and policy formulation standpoint, secondly from an operational and monitoring standpoint, and thirdly from a dynamic perspective, to identify the health gaps and implement effective and targeted policies for reducing health inequalities.

To the best of our knowledge this is the first study examining the effects of the economic crisis on the HRQoL of the young Greek population. The study highlighted the health gap in the EQ-5D-5L and EQ-VAS across age, sex, educational, and income groups. The international literature has highlighted the close relationship between economic crisis and anxiety/depression in young adults (53–55).

The prevalence of anxiety/depression and its association with various sociodemographic and mental health variables has been investigated by several researchers in large population-based studies for young Greeks. The results emphasized the impact of economic crisis on high prevalence of anxiety/depression among young adults (56–58).

In our analysis we investigated the effects of the economic crisis on anxiety/depression by specifying 4 models. The results of the empirical findings were presented in Tables 4, 5. In models 1 and 2 (see Table 4) “Before the crisis” the estimated coefficient for anxiety/depression were − 0.088 (*p* < 0.001) for the EQ-5D-5L and − 2.754 (*p* < 0.001) for the EQ-VAS. The effects of the crisis were investigated in the models 3 and 4 and the estimated coefficients presented higher values, i.e., −0.095 (*p* < 0.001) for the EQ-5D-5L and −3.164 (*p* < 0.001) for the EQ-VAS. The findings of our study support the results reached by other studies in Greece on the effects of the economic crisis on the anxiety/depression of the young people.

The empirical results of our study are also supported by other studies using the EQ-5D index as a measure of health inequalities. Low educational and income status is closely associated with lower EQ-5D health outcomes (59).

Our results are in line with previous research (60) highlighting the differences between EQ-5D-5L and EQ-VAS in measuring health related quality of life inequalities. The EQ-5D-5L appears to be a more sensitive indicator than EQ-VAS in capturing the magnitude of the health gap before and during crisis. In addition, Theil’s estimates on income related HRQoL inequalities before and during the crisis provided interesting results for health policy makers.

This study presents some strengths and limitations. Firstly, according to our literature review, one of the most noticeable strengths of our study, is the first one in Greece measuring HRQoL inequalities using the EQ-5D-5L instrument. Secondly, the large sample size of 4,177 young participants in our study from different demographic, and socio-economic backgrounds provides a strong base for statistical analysis. Thirdly, the measurement of health gap before (2009) and during (2016) the financial crisis in terms of the EQ-5D-5L and the EQ-VAS instruments. Fourthly, the employment of both between and within income related quality of life inequalities, measured by the Theil index. Fifthly, the useful results for effective and targeted public health policies aiming at the improvements of health and quality of life of young population in Greece.

However, some limitation of our study should also be seriously considered. Firstly, using the web-survey for only one wave in November 2015 to April 2016 and asking the subjects to recall their HRQoL in 2009 would bring a possible bias related to memory problems (recall bias). Theoretically, the best approach for investigating the dynamic effects on health-related quality of life is the longitudinal analysis. Nevertheless, the findings of other studies in Greece (57, 58) support the validity of our results, in measuring anxiety/depression and HRQoL during the economic crisis with the use of large-scale population studies. Similar method has been effectively used by the EuroQol group in Greece and in seven additional countries across the web reaching interesting results for European public health policies (61, 62).

Secondly, the assessment of several other economic, psychological, and mental health variables related to the effects of financial crisis upon the health and the quality of life of the young people is not included into our analysis (Confounding bias). Only an indirect reference was made to existing literature. Thirdly, collecting our data by a convenience sample in a web survey, possible limitation bias exists, because young population in remote geographical areas and lower socio-economic classes who have not access to computers on not being not familiar with digital questionnaires were excluded from our study.

Despite these limitations, the results of our study are useful for public health policies not only for Greece but also for the United Nations and the European Commission. The New Agenda of the United Nations for Sustainable Development (SDGs) focuses on 17 Millennium Goals to be attained by the year 2030 and invites the countries across the globe to adopt policies for the promotion of physical and mental well-being, universal health coverage, youth employment, fight against poverty, and the overall reduction in health inequalities. The findings of our research could possibly contribute to more targeted and effective National and European policies to reduce health inequalities among Nations Regions and Socio-Economic groups.

## Conclusion

Examining the literature of EQ-5D we have found limited applications of the instrument in depicting the effects of the crisis on populations’ health and quality of life. The purpose of this study was to cover the existing gap in the literature by launching a web-survey in Greece, which is a country with has experienced pretty dire consequences from the crisis in the economy and the health status of the population. The results of the present study highlight the significant impact of the crisis in the deterioration of the quality of life of the Greek people. The EQ-VAS mean score indicated a decline of subjective health by 9 points. In a similar vein, an even greater reduction was recorded in the EQ-5D-Index by 0.18 utility values. The EQ-5D-5L index appeared to be a more sensitive indicator to detect the effects of the crisis in comparison to EQ-VAS. The economic crisis appears to have a greater effect on Anxiety/Depression rather than the rest of the EQ-5D dimensions. On the basis of the findings of this study we may conclude that the EQ-5D-5L appears to be a sensitive instrument for measuring global health before and during the economic crisis and adequately depicting the deterioration of health-related quality of life among the poor and the less economically privileged population.

## Data availability statement

The raw data supporting the conclusions of this article will be made available upon request.

## Ethics statement

The studies involving human participants were reviewed and approved by the National and Kapodistrian University of Athens Ethics Committee approved the study. The patients/participants provided their written informed consent to participate in this study.

## Author contributions

JY conceived the study, designed the questionnaire, supervised data collection, and wrote the first draft of the manuscript. CA participated in the statistical analysis and drafting of the document. YP participated in the supervision of data collection, undertook the statistical analysis and participated in the writing of the report. All authors contributed to the design of the study. The content of this paper is not under consideration for publication elsewhere and all authors have read and approved the submitted version of the manuscript.

## Conflict of interest

The authors declare that the research was conducted in the absence of any commercial or financial relationships that could be construed as a potential conflict of interest.

## Publisher’s note

All claims expressed in this article are solely those of the authors and do not necessarily represent those of their affiliated organizations, or those of the publisher, the editors and the reviewers. Any product that may be evaluated in this article, or claim that may be made by its manufacturer, is not guaranteed or endorsed by the publisher.
